# Toward a Reversible Consolidation of Paper Materials Using Cellulose Nanocrystals

**DOI:** 10.1021/acsami.1c15330

**Published:** 2021-09-14

**Authors:** Alessandra Operamolla, Claudia Mazzuca, Laura Capodieci, Francesca Di Benedetto, Leonardo Severini, Mattia Titubante, Andrea Martinelli, Valter Castelvetro, Laura Micheli

**Affiliations:** † Dipartimento di Chimica e Chimica Industriale, Università di Pisa, via Giuseppe Moruzzi 13, I-56124 Pisa, Italy; ‡ Dipartimento di Scienze e Tecnologie Chimiche, Università degli Studi di Roma Tor Vergata, Via della Ricerca Scientifica, I-00133 Rome, Italy; § Unità CSGI (Consorzio Interuniversitario per lo Sviluppo dei Sistemi a grande Interfase) di Roma, Via della Ricerca Scientifica, I-00173 Rome, Italy; ∥ Laboratory for Functional Materials and Technologies for Sustainable Applications (SSPT-PROMAS-MATAS), ENEA − Italian National Agency for New Technologies, Energy and Sustainable Economic Development, S.S. 7 Appia km 706, I-72100 Brindisi, Italy; ⊥ Dipartimento di Chimica, Università degli Studi di Roma ″Sapienza″, Piazzale Aldo Moro 5 00185 Roma, Italy

**Keywords:** cellulose nanocrystals, FT-IR spectroscopy, nanotechnology, amperometric sensors, microscopy, cultural heritage, paper restoration, paper conservation

## Abstract

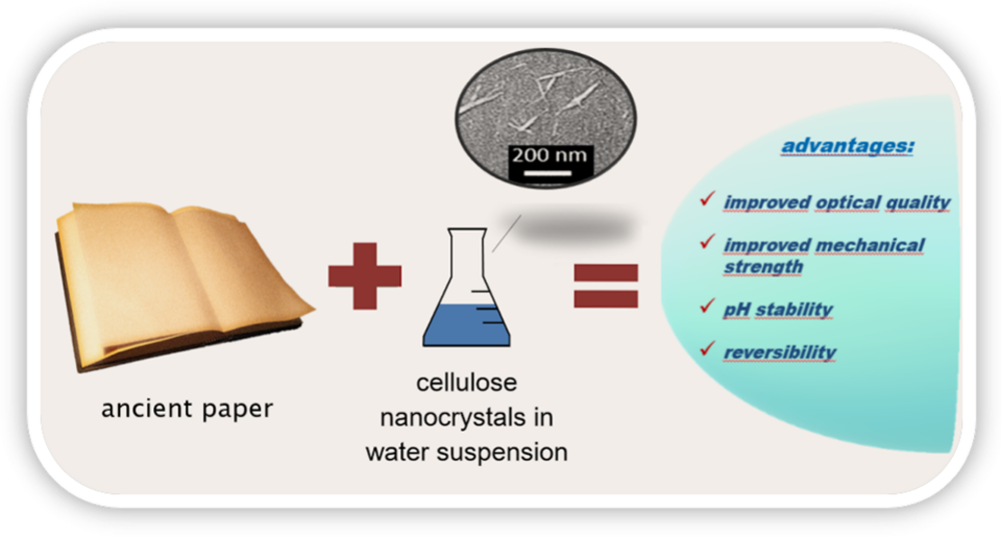

An innovative consolidation strategy for degraded paper is presented based on the reversible application of cellulose nanocrystals as sustainable fillers to reinforce mechanical properties and resistance to further degradation. The compatibility and efficacy of the proposed consolidation treatment are assessed first on pure cellulose paper, used as a model, by reliable techniques such as field emission scanning electron microscopy, atomic force microscopy, tensile tests, X-ray powder diffraction, and Fourier transform infrared spectroscopy, evidencing the influence of the surface functionalization of nanocellulose on the consolidation and protection effects. Then, the consolidation technique is applied to real aged paper samples from *Breviarium romanum ad usum Fratrum Minorum S.P.* (1738), demonstrating the promising potential of the suggested approach. Amperometric measurements, carried out with a smart electrochemical tool developed in our laboratory, demonstrate the reversibility of the proposed treatment by removal of the nanocrystalline cellulose from the paper surface with a suitable cleaning hydrogel. This completely new feature of the consolidation treatment proposed here satisfies a pivotal requisite in cultural heritage conservation because the methodological requirement for the ″reversibility″ of any conservation measure is a fundamental goal for restorers. A paper artifact, in fact, is subject to a number of natural and man-made hazards, inducing continuous degradation. With time, monitoring and consolidation actions need to be often performed to ensure conservation, and this tends to modify the status quo and compromise the artifact integrity. Removable treatments can potentially avoid erosion of the artifact integrity.

## Introduction

Precious and degraded paper artworks necessitate restoration treatments to preserve their unmeasurable artistic and historical value and to slow down irreversible degradation processes. In this context, the consolidation of very fragile artworks often represents a necessary, but delicate step. Typically, the strengthening of fragile papers is performed by the use of linings. This method could have several disadvantages because it involves the use of: (i) an external (other than artwork itself) paper material with different properties with respect to the original one and (ii) a glue. With aging, some of these additional materials undergo structural transformations that could cause a loss in compactness, yellowing, and an acidity increase, accelerating the degradation processes of the artwork itself.^1−4^ Only recently several innovative and sustainable methods, involving the use of nanocelluloses of different origin, crystallinity and aspect ratio, have been pointed out to consolidate fragile biomaterials like canvas,^5,6^ and, in just very few studies, wood^7^ and paper artworks.^8−10^ Indeed, nanocelluloses display a high affinity for the cellulose fibers as in canvas and paper because they are composed of the same biomaterial, cellulose, and do not require the use of any adhesive for their application. The crystalline nanocelluloses are the ideal materials for restoring the original properties of paper. In a few attempts reported in the literature, bacterial nanocellulose (BNC) or cellulose nanofiber (CNF) thin films were applied by conventional lining (i.e., attachment to a paper surface) on the deteriorated paper with encouraging results that well compare to the ones given by the more traditional Japanese paper.^8−10^

However, some issues of the above-described treatments with CNFs and BNC are still open, mainly pertinent to the durability of the treatment and demonstration of its reversibility.

Nanocrystalline celluloses are abundant and nontoxic.^11−13^ These nanomaterials display exceptional mechanical^14−16^ and thermal^17^ properties, optical transparency,^18^ and high crystallinity^19^ and have extensive application in many fields of chemistry and chemical engineering.^20−27^ Cellulose nanocrystals (CNCs, high aspect ratio crystalline structures with a diameter ranging from 5 to 50 nm and length between 100 and 500 nm)^28^ are often used as fillers and reinforcing agents in nanocomposites^29^ and as paper pulp modifier to increase its resistance or control its water absorption.^30^ CNCs are highly efficient nanocomposite formers: for example, they can be dispersed at high concentration in water and applied on a canvas surface. In this specific application, nanocelluloses differing in dimension and chemical functionalization manifest diverse consolidation effects, with the highest reinforcement per equivalent number of coatings yielded by CNC treatment with respect to nanofibers with a higher aspect ratio.^5,6^ Moreover, they form the most homogenous film which best protects canvases from mechanical changes upon relative humidity variations.^6^ CNCs mixed to silver nanoparticles can protect Whatman paper from microbial attacks.^31^ Finally, an interesting study from the University of Florence on the application of oleic acid-treated CNCs combined with calcium carbonate nanoparticles for consolidation of artificially aged filter paper as a model of ancient paper artworks has further highlighted the potential of nanocellulose coating for paper preservation.^32^

Given the above-described scenario and in connection with our previous studies,^3,33−37^ in the present work, the use of neutral and sulfated CNCs in paper artwork conservation treatments is presented, using pure cellulose paper samples (Whatman paper) as a test. The nanocrystals used in our study differ in their surface functionalization: while N_CNCs are composed of pure cellulose, S_CNCs present surface derivatization with sulfate groups, which may influence their behavior once they are applied on paper. Therefore, in our work, we offer an important highlight on the influence of the surface functionalization of CNCs on their behavior as a paper consolidant. Given the positive outcomes in terms of paper characteristics, we extend the study to a written real aged paper sample from the XVIII century (*Breviarium Romanum ad usum fratrum minorum*), a rag pulp book previously characterized in our laboratories.^38−40^ Finally, we propose here a solution to a pivotal issue of restoration processes,^41^ demonstrating for the first time that the treatment with CNCs is reversible by their easy and safe removal via gellan gel application.^3,38^ Indeed, degraded artworks need that measures are taken often to ensure the conservation, and in some cases, removal of previous consolidating materials can erode artifact’s integrity. Therefore, herein, we demonstrate that they can be safely removed by paper treatment with a gel.

In order to offer a thorough and quantitative evaluation of the consolidation method, experiments using different techniques have been performed on paper samples before and after the treatment with CNCs: field emission scanning electron microscopy (FE-SEM), atomic force microscopy (AFM) topographies, elemental analysis, tensile tests, colorimetry, pH measurements, and Fourier transform infrared spectroscopy (FTIR). Each of them gives complementary information on the goodness of CNCs as reinforcement materials for paper artworks.

## Experimental Section

### Materials

Pure cellulose paper samples (Whatman filter paper N° 1) were obtained from Whatman (Maidstone UK). Gellan gum was a KELCOGELCG-LA product obtained by CP Kelco (Atlanta Georgia, USA). *Cellulase* [(≥ 0.3 U/mg; EC 3.2.1.4 from *Aspergillus Niger*)], D (+)-glucose monohydrate, glucose oxidase, Avicel PH 10.1, and sulfuric and hydrochloric acid were obtained from Merck (Merck KGaA, Darmastadt, Germany, Europe). All reagents were of analytical grade and used without further purification. In the preparation, solutions of bidistilled water (Millipore, Merck, KGaA, Germany) was used. The book “*Breviarium Romanum ad usum fratrum minorum*” (called, in the following, *Breviarium*) of 1738 is from a private collection.

### Preparation of Sulfated CNCs

This procedure was adapted from the work by Operamolla et al.^42^ where 40 mL of deionized water was introduced in a 250 mL three-necked round-bottomed flask equipped with a water condenser and a mechanical stirrer. Then, the flask was cooled in an ice bath, and 40 mL of concentrated H_2_SO_4_ was added. After that, 4 g of Avicel PH-101 was added, and the suspension was warmed to 50 °C for 80 min. The system was cooled to room temperature, and the mixture was transferred to polypropylene centrifugation tubes. Centrifugation at 4000 rpm was repeated, replacing the supernatant liquid with fresh deionized water until the pH was approximately 1. Then, the precipitate was suspended in deionized water with the aid of a Branson sonifier 250 (Danbury, CT) equipped with an ultrasonic horn with a 3.5 mm diameter (micro-tip) operated in pulsed mode, with a power of 40 W, 0.6 s pulses for 10 min, and dialyzed against distilled water until neutrality using a cellulose nitrate membrane with a molecular weight cut-off of 12,400 Da. The resulting suspension was transferred to polypropylene centrifugation tubes and centrifuged at 4000 rpm for 20 min. The supernatant suspension was kept, and water was removed under reduced pressure, yielding 905 mg of CNCs with an average length of 280 ± 70 nm.

### Preparation of Neutral CNCs

Fifty milliliters of deionized water was introduced in a 250 mL three-necked round-bottomed flask equipped with a water condenser and a mechanical stirrer. Then, the flask was cooled in an ice bath and 50 mL of concentrated HCl was added. After that, 5.00 g of Avicel PH-101 was added and the suspension was warmed to 105 °C for 6 h. The system was cooled to room temperature and diluted with 50 mL of distilled water and the mixture was transferred to polypropylene centrifugation tubes. Centrifugation at 3000 rpm was repeated four times, replacing the supernatant liquid with fresh deionized water until the pH was approximately 3. Then, the precipitate was dialyzed against distilled water until neutrality using a cellulose nitrate membrane with a molecular weight cut-off of 12,400 Da. The resulting suspension was recovered, and water was removed by distillation under reduced pressure, yielding 4.71 g of CNCs.

### Paper Sample Preparation

Sonication of water suspensions was carried out with a Branson Sonifier 250 used at 40 W power in the case of S_CNC and at 80 W power for N_CNC. A total of 1.5 mL of 1.5% of sonicated suspension of N_CNC or of 2% suspension of S_CNC was applied by a soft brush on stripes of Whatman or *Breviarium* paper samples of 8.0 cm^2^ dimensions. After drying, the resulting weight increase (%) of treated paper is in accordance with the amount of CNCs added (0.4 and 0.3% after N_CNC and S_CNC, respectively). The results obtained for these CNC-treated samples were compared, Whatman and Breviarium paper treated with only pure water, applied with a soft brush (called, in the following, as pristine paper sample). We have decided to compare the data of CNC-treated paper samples with those treated with pure water because all the specimens have undergone treatment with water (with or without CNCs), thus rendering the results clearer and making the effect of CNCs more evident.

Paper aging on untreated and treated paper samples was performed, following the procedures reported by Lojewska et al.^43^ and Calvini et al.:^44^ paper samples were immersed for 1 h in a solution of 0.1 M HCl to catalyze hydrolysis and then aged for 1 week at 80 °C and RH = 65%.

### Atomic Force Microscopy

AFM topographies were carried out using a Park XE-100 SPM system microscope. Images were acquired in the tapping mode using tips (Type PPP-NCHR) on a cantilever of 125 μm length, about 330 kHz resonance frequency, 42 N m^–1^ nominal force constant, and 10 nm guaranteed tip curvature radius. Surface areas were sampled with a scan rate of 1 Hz. The topographies were analyzed using the software XEI (Park System Corporation, version 1.8.0). For the AFM analyses of CNCs, 15 mg of sulfated or neutral samples was dispersed in 5 mL of distilled water with the aid of a tip sonicator (power 40 W, duty cycle 60%, time 6 min), and the resulting suspension was cast on mica and allowed to dry in ambient conditions.

### FE-SEM Images

The morphology of the surface of paper samples was analyzed by FE-SEM on a field emission scanning electron microscope ZEISS Merlin equipped with a GEMINI IIs column and Beam-Booster, with acceleration voltages between 0.05 and 30 kV and 0.8 nm as the best resolution, four optional detectors for SE and BSE, charge compensation, and an in situ sample cleaning system. 1 × 1 cm^2^ quartz glasses or silicon slabs were used as substrates for FE-SEM measurements. All samples were dried in vacuum before the analysis.

### Glancing Incidence X-ray Diffraction

The surface-sensitive structural analysis was performed by using an X-ray diffraction (XRD) system (EMPYREAN, PANalytical) equipped with a parallel beam mirror (incident optic) coupled with a diffracted beam collimator (equatorial acceptance of 0.27°). In a typical measurement, the spectra are recorded by maintaining the incident angle ω (angle between the incident beam and sample surface) fixed at 1.5° and moving the X-ray detector and post-sample collimator along the goniometer circle in the 2θ range between 5 and 45° with a step size of 0.02°. Where necessary, the crystallinity index (C.I.) of cellulose was calculated from the XRD spectra by the method reported by Segal,^45^ according to the following equation
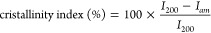
1where *I*_200_ represents the maximum intensity of the peak with Miller’s indexes 200 (centered at 22.6° in our spectra), while intensity of the amorphous peak is calculated at the maximum, which depends on the typology of cellulose and is centered at 18° for cellulose I and 16° for cellulose II.

### Colorimetric and pH Experiments

Colorimetric measurements were performed using a Minolta operating in the D65/10 mode. Results were expressed in CIELab color space (L, a*, b*), L* for the lightness from black (0) to white (100), a* from green (−) to red (+), and b* from blue (−) to yellow (+). An average of six measurements was performed for each colorimetric value.^38^ CIELab is a three-dimensional space, and the distance (expressed numerically with Δ*E*) between two color coordinates can be calculated by applying the Pythagorean Theorem. This value is useful to interpret the meaning of the psychrometric differences in color (only if Δ*E* > 3 color difference can be detected by human eyes).^46^ Variation of the colorimetric values are calculated with respect to those obtained using the pristine paper sample.

Measurements of pH were carried out on the paper surface by using an Amel Instrument 334-B pH meter with a combined glass electrode Ag/AgCl and a porous PTFE diaphragm (Crison Instruments, Spain); relative standard deviation was 5%, calculated on three measurements of the same sample.

### FTIR Analyses

Spectra were acquired on a ThermoScientific (mod. Is50) instrument, equipped with an attenuated total reflectance (ATR) diamond cell for measurement in the 4000–525 cm^–1^ region. A total of 64 scans were collected at a resolution of 4 cm^–1^ for each sample. ATR-FTIR spectra were recorded in triplicate.

### ζ-Potential Measurements

The ζ-potential of CNC in water could only be determined for the colloidally stable S_CNC dispersion. The measurements were performed with a Brookhaven 90 Plus Dynamic Light Scattering particle size analyzer equipped with ZetaPlus software and electrophoretic light scattering cell for determination of the ζ-potential. The measurements were performed on a sonicated 10 mg·L^–1^ S_CNC dispersion in 1 mM aqueous KCl at pH ∼ 7.0 without addition of any surfactant.

### Elemental Analyses

Elemental analyses were performed on a Carlo Erba EA 1108 CHNS Elemental analyzer or on an Elementar Vario Micro Cube analyzer. Sample preparation: paper samples were cut into different pieces, dried in vacuum, and weighed on a precision balance, placed inside tin caps, and analyzed immediately afterward. Samples weight was maintained between 2.0 and 3.3 mg, except for analyses of S_CNCs and N_CNCs, for which the sample weight was ∼5 mg to ensure detection of small amounts of elements (S or N).

### Tensile Tests

Tensile test on paper specimens, with dimensions 165 × 15 mm, have been performed by means of a universal testing machine (Instron 4502, NJ, USA*),* following the UNI EN ISO 1924-2:2009 standard. A gauge length of 125 mm has been set. Eight repetitions for each sample have been performed.

### Monitoring Tool Coupled with the Electrochemical Biosensor

The tool (Figure S1) is composed of a flow sampling plate in contact with a hydrogel (Gellan gel). The flow sampling plate is connected to an electrochemical biosensor, sensitive to d-glucose. The biosensor and the preparation of Gellan gel and its application on the paper surface have been already described.^3,34,38^ Briefly, the flow sampling system is applied directly on the Gellan gel on the opposite side with respect to the paper surface and is constituted by a plate in Perspex with a serpentine with 12 channels. Through the serpentine, the buffer flows continuously (moved by a peristaltic pump MINIPLUS3—Gilson, USA), lapping the gel applied on paper samples (Figure S1). All the material removed by the gel from the paper sample is taken and dragged away, through the flow tubes, to a bioreactor where the enzymatic degradation of nanocellulose (see below) occurs. Then, the material is dragged into an electrochemical thin layer cell, where a screen-printed biosensor is integrated. The thin layer cell is a liquid chromatography–electrochemistry cell obtained from Bio-Analytical Systems (West Lafayette IN, USA), adapted for screen-printed electrodes (SPEs). The working and carrier buffer was 0.05 M phosphate buffer +0.1 M KCl, pH 6.8. The buffer flow (0.1 mL/min) was regulated by a peristaltic pump, connected directly to the plate. Amperometric measurements were carried out using a PalmSens potentiostat (Palm Instruments BV, The Netherlands) connected to a laptop computer.

SPEs were printed in the Laboratory of Analytical Chemistry of the University of Rome Tor Vergata. The diameter of the working electrode was 0.3 cm, resulting in a geometric area of 0.07 cm^2^. To obtain biosensors, the SPEs were modified with Prussian Blue (PB) [potassium iron(III) hexacyanoferrate(II)] prior to enzyme immobilization. The procedure of the working electrode surface modification protocol was reported in a previous work.^34^

The bioreactor for nanocellulose analysis was a hand-made cylindrical reactor in Plexiglass in which an enzymatic ABC Immunodyne membrane (Pall Corporation, Ireland) was inserted into the flow system. The membrane had an immobilization surface of 300 cm^2^, with a thickness of 150 μm. A total of 13 μL of enzyme solution [200 IU ml^–1^*Cellulase* (≥ 0.3 U/mg; EC 3.2.1.4 from *Trichoderma sp* and *Aspergillus niger*)] in 0.1 M phosphate buffer (pH 8.0) was added on both membrane faces and incubated for 1 h at room temperature. The gel was applied on paper samples for 1 h. This electrochemical device is able to produce a current proportional to the amount of glucose present in the solution flow, yielding a real-time efficient monitor tool.

## Results and Discussion

### Preparation and Characteristics of Sulfated and Neutral CNCs

CNCs were prepared by hydrolysis with strong mineral acids^47,48^ at controlled reaction conditions. Sulfuric acid hydrolysis yielded easily suspendable negatively charged (at pH > 1) CNCs,^42^ named S_CNC (sulfated CNC), due to a parallel process of surface sulfation, randomly involving the primary hydroxyl groups on the surface glucopyranose units.^49^ S_CNCs formed stable colloidal suspensions in water at 2% (w/w) concentration,^50−52^ thanks to surface charge repulsion. Conversely, hydrolysis conducted with hydrochloric acid produced uncharged nanocrystals, named N_CNC (neutral CNC), which displayed higher aggregation tendency, and were suspended in distilled water at 1.5% (w/w) final concentration.^53^

**Figure 1:**
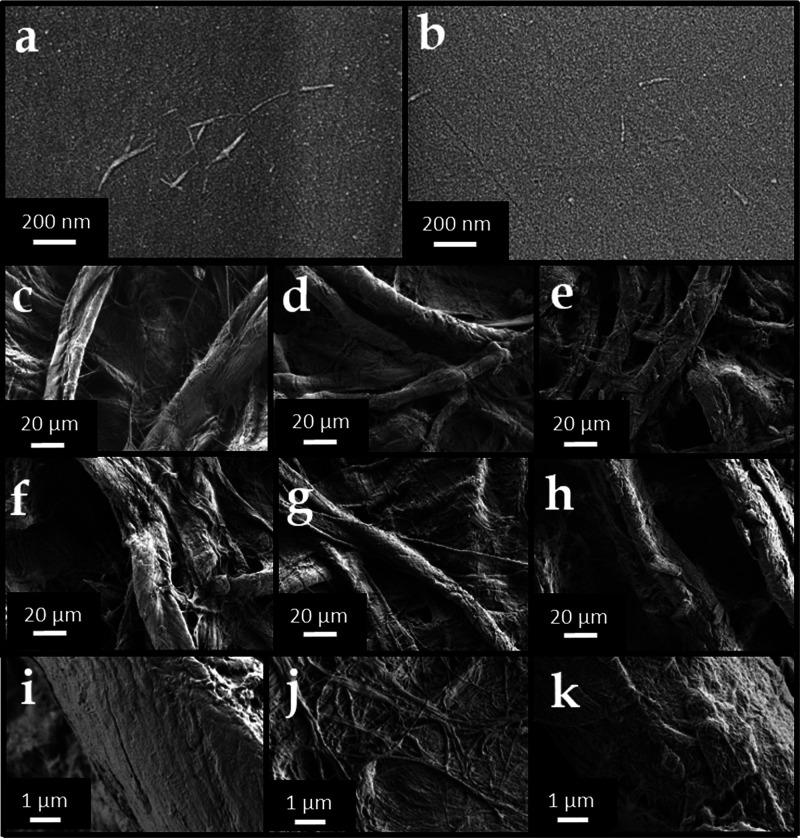
FE-SEM micrographs of (a) S_CNCs deposited on silicon from 10 mg L^–1^ water suspension and (b) N_CNCs deposited on silicon from 1 mg L^–1^ DMSO suspension, scale bar 200 nm. Panels c–e show FE-SEM micrographs of (c) pristine Whatman paper; (d) Whatman paper after application of S_CNCs; and (e) Whatman paper after application of N_CNCs, scale bar 20 μm. Panels (f–h) display FE-SEM micrographs of samples presented in panels (c–e) after accelerated aging: (f) pristine Whatman paper after accelerated aging; (g) Whatman paper after application of S_CNCs after accelerated aging; and (h) Whatman paper after application of N_CNCs after accelerated aging; scale bar 20 μm. Panels (i–k) show the same samples as in (c–e) at higher magnification: (i) pristine Whatman paper; (j) Whatman paper after application of S_CNCs; and (k) Whatman paper after application of N_CNCs; scale bar 1 μm.

The two nanocellulose forms were investigated by AFM and FE-SEM to detect their morphology and dimension. While AFM topographies of thin films of S_CNCs and N_CNCs (reported in Figure S3) demonstrate the good filming ability of both, the FE-SEM investigation revealed average lengths of 149 ± 32 nm and 151 ± 17 nm for S_CNCs and N_CNCs, respectively. In both cases, the thickness of the nanocrystals was detected by AFM to be 10 ± 2 nm. These data are collected in Table 1. Figure 1 panel (a) shows a SEM micrograph of S_CNCs deposited on silicon (111) from water suspension at a concentration of 10 mg L^–1^. These nanocrystals showed the tendency to form small clusters connecting to each other by the long side. Panel (b) shows N_CNCs deposited on the same substrate from dimethyl sulfoxide (DMSO) at a concentration of 1 mg L^–1^. Apparently, N_CNCs looked very similar in morphology to S_CNCs, but their dispersing ability in water was much poorer, as we could not detect separate N_CNCs deposited from water but only clusters or films. The quite analogous length and thickness displayed by individual nanocrystals of S_CNCs and N_CNCs should be related, more than the kind of acid used for the digestion, to the cellulose source (Avicel) and purification protocol applied. The quite different dispersing properties, shown by S_CNCs and N_CNCs in water, were attributed to the different surface charges possessed by the two nanocellulose samples: ζ-potential measurements were performed on nanocrystals suspensions, revealing for S_CNCs an observed ζ-potential value of −28.3 ± 1.3 mV at pH 7.0. Such negative surface charge endows the aqueous dispersions of sulfated CNCs^54^ with a fair colloidal stability as opposed to the case of N_CNCs. We also performed elemental analyses on both S_CNCs and N_CNCs for detecting the degree of substitution with sulfate groups on S_CNCs. As expected, N_CNCs revealed the absence of sulfur. Conversely, S_CNC revealed the following composition: C% 40.35, H% 5.89, S% 0.88, and O% 52.88. For the calculation of the cellulose degree of substitution (i.e., the molar number of sulfate groups for each glucopyranose ring), we elaborated the results of elemental analyses, as described in the Supporting Information. S_CNCs were composed of pure cellulose with a substitution degree of sulfate groups of ∼0.05. The water content calculated from elemental analyses, as well as the value of pH found for the relevant S_CNC suspensions (immediately after dialysis and before freeze-drying) of 3.94, would suggest that sulfate groups and H_3_O^+^ ions were co-present in the freeze-dried sample.

**Table 1:** Characterization Data of Cellulose Nanocrystals Used in This Work

				elemental analyses				
sample	average length [nm]	average thickness [nm]	ζ potential [mV]^a^	C %	H %	S %	O %^b^	pH^c^
S_CNC	149 ± 32	10 ± 2	–28.3 ± 1.3	40.35	5.89	0.88	52.88	3.94
N_CNC	151 ± 17	10 ± 2		40.83	6.52	0.00	52.65	6.60

a : ζ potential was measured in 1 mM KCl_aq_ at pH 7.0. The concentration of the nanocrystals was approximately 10 mg L^–1^.

b Oxygen weight percentage was calculated by difference.

c The pH was measured on the CNC suspension after dialysis and prior to freeze-drying.

Other different properties could be expected on the basis of other literature reports,^54−57^ such as (in some cases dramatically) lower thermal stability for sulfated nanocellulose with respect to the corresponding less dispersible neutral form, which is a relevant property, considering the conditions that were used in our artificial aging experiments on paper samples (see below). The reported lower decomposition temperatures were ascribed to the easier elimination reaction of sulfate groups from the glucopyranose units, with respect to the elimination of a molecule of water, which requires more energy. These findings, combined with an observed increase of char content, hinted at a fire-retardant role of the sulfate moieties. The behavior was observed even at very low degrees of functionalization with sulfate groups. This is the reason why both nanocellulose forms and not just the most common sulfated one were judged worth investigating: in this way, we aimed at providing a benchmark study on consolidation treatments performed with the nanocelluloses, including useful new information about the effect of nanocellulose surface functionalization on the restoration success. Indeed, aging experiments performed on treated and untreated paper helped, in our case, to understand the role of sulfate groups on the preservation of paper artworks.

XRD spectra acquired on pure N_CNCs and S_CNCs thin films are shown in Figure S2. The spectra revealed the presence of much purer cellulose I phase in N_CNCs. Conversely, S_CNCs displayed the still predominant presence of cellulose I phase, but with higher contamination from amorphous or cellulose II phases, as shown by the characteristic diffraction peaks in the region between 19 and 21 θ, indicating the influence of the sulfuric acid hydrolysis reaction on the phase composition of the starting crystalline cellulose.

### Characterization of Whatman Samples with CNCs before and after Aging

Sulfate and neutral CNC aqueous dispersions were applied on new and artificially aged Whatman paper samples. Paper aging on paper samples was performed by a modified literature procedure, as detailed in the Experimental Section. The dispersions containing the consolidants were manually applied on paper with a brush in order to reproduce the same effect as in a real case of conservation treatment procedure; after drying, accurate morphological, mechanical, and chemical analyses were performed.

FE-SEM micrographs of pristine Whatman paper and the same paper treated with S_CNCs or N_CNCs are given in Figure 1(c–e) panels and at the highest magnification in Figure 1(i–k) panels. Figure 1c displays the morphology of the pristine paper sample (unaged Whatman paper), for comparison, while the artificially aged Whatman sample is shown in Figure 1(f). Whatman paper treated with S_CNCs and N_CNCs is shown in Figure 1(e,f), respectively. CNCs were in both cases deposited on cellulose fibers, covering the samples; importantly, while N_CNCs displayed the tendency to coat individual paper fibers forming a network on their surface (Figure 1(k)), S_CNCs formed films apparently filling the pores among cellulose fibers. This feature is clearly visible by comparing the micrographs presented in Figure 1(d,e), and it suggests that the presence of functional groups on CNCs influenced their adhesion to paper fibers and their film-forming ability.

**Figure 2:**
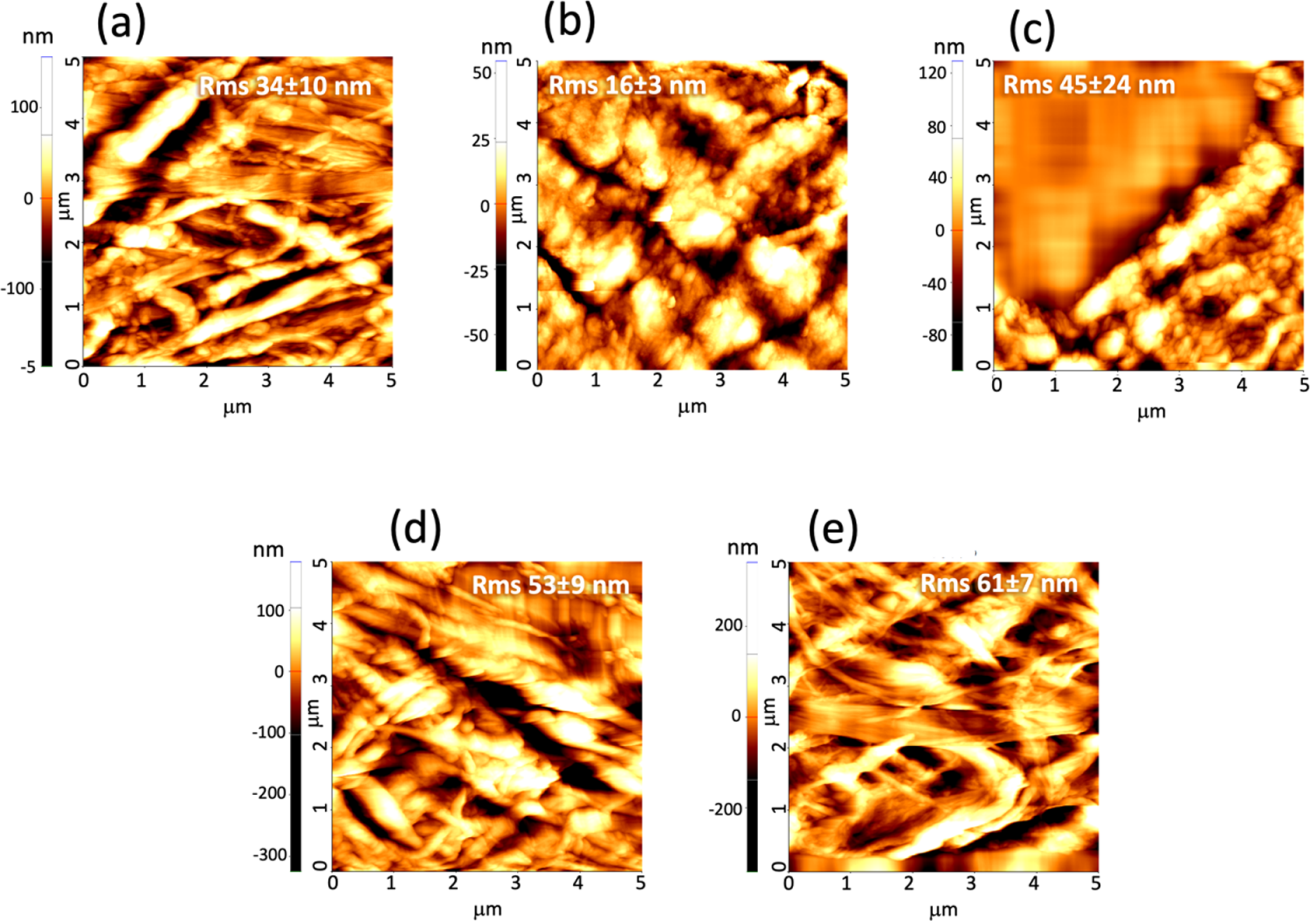
5 × 5 μm^2^ noncontact mode AFM topographies of (a) untreated Whatman paper; (b) paper treated with S_CNCs; (c) paper treated with N_CNCs; (d) Whatman paper treated with S_CNCs after cleaning with Gellan gel; and (e) Whatman paper treated with N_CNCs after cleaning with Gellan gel. Inside the topographies, the value of the root mean roughness in nm is reported.

In addition to FE-SEM, we performed an investigation of paper samples by AFM in noncontact mode: the AFM results, indeed, provided a more accurate identification of nanostructures with respect to SEM combined to information on surface roughness. The noncontact mode was preferred to avoid damages to the surface of paper. The topographies are shown in Figure 2, panels (a–c). 5 × 5 μm^2^ scanning areas allowed us to acquire surface morphologies, where paper fibers and CNCs could be easily distinguished. We preliminarily investigated the following samples: (a) pristine Whatman paper; (b) Whatman paper treated with S_CNCs; and (c) Whatman paper treated with N_CNCs. The pristine paper (Figure 2(a)) presented bundles of fibers whose surface could seldom appear as nanostructured, with a surface roughness of ∼34 ± 10 nm. The texture of the paper samples covered with S_CNCs and N_CNCs consistently changed with respect to the starting paper, as aggregates of nanoparticles appeared in both samples, hiding the underlying paper structure (Figure 2(b,c)). Results obtained from this technique confirm that the coverage effect of the charged and uncharged CNCs is quite different; while treatment with N_CNCs (Figure 2(c)) increased surface roughness at a point, in some regions, the cantilever drift recorded huge flat valleys (clearly an artifact of the technique), yielding an increased average surface roughness of 45 ± 24 nm. The treatment with S_CNCs decreased the roughness value to ∼16 ± 3 nm (Figure 2(b)). This behavior strongly suggested the ability of S_CNCs to spontaneously organize into self-assembled thin films that covered the paper surface more homogeneously. Conversely, N_CNCs apparently bound strongly to the surface of individual paper fibers without forming self-standing films. Based on observation, we cannot rule out a possible partial penetration of N_CNCs into the paper structure corresponding to the pores among the fibers,^31^ as discussed later, on the basis of the mechanical tests. The behavior of N_CNCs resulted, overall, in an increased roughness, which pointed at an increased thickness of paper fibers.

The three samples described above were also analyzed by XRD measurements, which were performed under glancing incidence conditions, to enable the identification of phases with increased surface sensitivity. The XRD spectra acquired on the paper samples are displayed in Figure S4. The spectra revealed that the three samples were mainly composed of cellulose Iβ with a limited presence of amorphous components, as expected for samples consisting of Whatman paper, where the nanocellulose mass fraction had negligible influence on the relevant spectra. Indeed, in spite of the different functionalization of the two starting nanocellulose materials, only small variations of diffraction intensity were recorded in the collected spectra.

This finding was supported by the invariance in the C.I. (that is, the ratio of the intensity of the band localized at 2900 and 1370 cm^–1^) obtainable from the analysis of the FTIR spectra (see Figure S5 and Table 2). As expected, the FTIR spectra of the three samples presented very similar features, and the C.I. remained almost constant, varying from 0.64 ± 0.03 (pristine samples) to 0.65 ± 0.03 (for samples treated with N_CNCs or S_CNCs).^58^ The invariance on results can be easily explained by the fact that the amount of CNCs added on a paper sample is significantly low (the weight increase of Whatman paper is about 0.3% after CNC treatment), so that it does not perturb significantly the overall crystallinity properties of paper. Macroscopically, the possible perturbation on the optical quality of paper after application of CNCs was evaluated by performing colorimetric experiments. These experiments are more reliable than a simple human eye inspection, which is insensitive to yellowing or darkening of paper if the colorimetric change ΔE* (in terms of lightness and chromaticity values ΔL*, Δb*, and Δa*) is lower than 3.^45,46^ These parameters are reported in Table 2. Application of CNCs (both typologies) caused only a negligible increase of lightness and a variation of a* and b* values [a* varies from red (+) to green (−), while b* varies from yellow (+) to blue (−)]. Moreover, pH, a fundamental parameter in evaluating the health state of a paper material,^45,46^ did not change substantially upon treating the samples with CNCs. Conversely, the mechanical properties of the samples coated with CNCs slightly improved, suggesting that CNCs increase, even if slightly, the tensile strength of paper. This result is in contrast with other literature reports, where the negative influence of the mechanical coating with nanocellulose water suspensions on paper properties was observed due to the forced penetration of water into the fibrous structure of paper;^59^ it is in agreement with the results reported by Bergamonti et al.,^31^ indicating the positive effect of the manual application with a brush on paper properties, which will not force water to penetrate deeply among paper fibers.

**Table 2:** Colorimetric Variations (Δ*E*), pH, FTIR Crystallinity Index Values, and Results of Tensile Tests of the Whatman Paper Samples under Investigation

paper	Δ*L*	Δ*a*	Δ*b*	Δ*E*^a^	pH	crystallinity index [C.I.]^b^	maximum stress [MPa]	maximum strain [%]
pristine					6.7 ± 0.2	0.64 ± 0.03	23 ± 1	1.7 ± 0.3
with S_CNC	0.13	–0.09	0.07	0.17 ± 0.5	6.4 ± 0.2	0.65 ± 0.03	23 ± 1	2.1 ± 0.2
with N_CNC	0.12	–0.07	0.03	0.14 ± 0.5	6.5 ± 0.2	0.65 ± 0.03	25 ± 1	2.0 ± 0.1
aged	0.19	–5.36	4.91	7.27 ± 0.05	5.7 ± 0.2	0.59 ± 0.03	12 ± 1	1.7 ± 0.3
with S_CNC and aged	0.39	–5.33	5.48	7.65 ± 0.09	4.5 ± 0.2	0.64 ± 0.03	18 ± 1	2.1 ± 0.5
with N_CNC and aged	0.21	–5.38	5.25	7.52 ± 0.07	5.7 ± 0.2	0.64 ± 0.03	15 ± 4	1.7 ± 0.3

a Variations are referred to a reference value measured for the pristine sample.

b C.I. measured from FTIR shows the same trend as C.I. measured from XRD. Additional details are given in Table S1

Once the preliminary investigation showed that coating with S_CNCs and N_CNCs does not perturb or deteriorate significantly the optical and mechanical quality of paper samples and indicated the potentialities of improving paper characteristics, we subjected the paper samples treated with the nanocelluloses to a process of artificial aging, with the aim of testing their morphological, pH, chromatic, and mechanical stability. This investigation allowed us to assess the real protective potential of the two treatments against degradation of the paper samples, a necessary requisite for a consolidation technique. These data are shown in Figure 1(f–h) and Table 2 (last three rows).

The morphological stability of the coating was demonstrated by comparing FE-SEM micrographs acquired on the samples restored with both typologies of CNCs after the aging treatment (Figure 1(g,h)). The C.I. obtained by FTIR measurements on aged samples treated with CNCs was 0.64 ± 0.03, which is slightly higher than that observed for the pristine Whatman sample (0.59 ± 0.03). It should be noted that these differences are significant, considering the low amount of CNCs added (about 0.3% of total weight). The stability of C.I. values observed on Whatman paper samples treated with CNCs after aging indicated a remarkable stability of CNC coating, suggesting their protective ability toward water or oxygen contamination (chemical agents mainly responsible of paper degradation in the experiments carried out) of paper fibers. Considering the thickness sampled in ATR mode (∼2 μm), the C.I. measured referred mainly to paper material, rather than the negligible amount of nanocellulose coating, as explained earlier. Moreover, the treatment with CNCs did not compromise the optical quality of samples even after aging, as the colorimetric variations were the same for the treated and untreated aged samples. These results, reported in Table 2, suggested that the proposed restoration method is suitable for paper samples. However, a more detailed inspection showed that S_CNC-treated and N_CNC-treated paper samples aged differently. The pH values revealed a dramatic difference between the two aged samples. The paper treated with S_CNCs showed a more dramatic decrease of pH toward an acid value (from 5.7 to 4.5, see Table 2), while treatment with N_CNCs did not alter the pH value of paper. This result was in agreement with data recently reported by Zhang et al. on the aging of nanopaper^60^ and those obtained by a study performed by the University of Florence, which proposed the use of a deacidifying additive together with sulfated nanocellulose to raise the pH of paper.^32^ Aging of Whatman paper can be related to oxidation phenomena, with the appearance of carboxyl groups increasing the acidity content.^61^ We may speculate, based also on the results reported by Zhang et al., that an early elimination of the sulfate group as hydrogensulfate, catalyzed by the acid compounds (such as HCl, used in the artificial aging experiments, but, in real samples, those from inks, pollutants, and organic acids arising from cellulose degradation^43,44^), accelerates the decrease of pH, showing the worsening effect on pH of the oxidation of C6 to carboxyl groups, but the point remains open to further investigation.

Finally, it should be noted that the effect of this preservation treatment on the mechanical properties of aged paper samples is very promising. In spite of the very low nanocellulose content (about 0.3% by weight), tensile strength values, reported in Table 2, revealed a significant improvement of maximum stress and maximum strain values of S_CNC- and N_CNC-treated paper, with respect to aged and unprotected Whatman paper. This finding suggests that the adsorption of CNCs on paper fibers reinforced them and their interconnection, demonstrating the goodness of the proposed restoration method. In this case, better results were obtained with S_CNCs, indicating that the presence of charged residues positively influenced the interconnection among cellulose chains through the establishment of polar interactions.

Elemental analyses (see Table S3), performed on the pristine and treated samples, evidenced in pristine Whatman paper the presence of humidity traces, as witnessed by the lower carbon content with respect to the theoretical carbon percentage expected from pure cellulose (41.88 vs 44.45% theoretical value). An increase in carbon content was observed with the aging process for all samples (from 41.88% for Whatman paper to 42.48% for Whatman paper after aging). The higher percentage of carbon was retained after treatment with S_CNCs and lowered after N_CNC treatment. We took this observation as a confirmation of a different behavior of neutral or charged CNCs, probably connected to loss of sulfur due to desulfation processes occurring on the S_CNC with aging. However, elemental analyses could not give us any information on the amount of eliminated sulfur because its overall content was too low when compared to the detection limit of the technique.

### Reversibility of the Conservation Treatment with CNCs

The interesting aspect and true novelty of the restoration of paper performed by the application of a protecting coating with CNCs is the reversibility of the treatment, a capability that we are able to demonstrate. To ensure this reversibility, we used a cleaning method, based on the application of a Gellan hydrogel on the paper surface;^3,4,33,34,37,38,40^ the other surface of the gel is coupled with a flow sampling plate, in line with an electrochemical system designed ad hoc for sensing the amount of cellulose eventually removed from the paper surface.^3,34,37,38^ Gellan hydrogel is a Gellan gum-based hydrogel, widely used for the cleaning of paper artworks.^3,4,33,34,37,38,40^ During the cleaning process, it can be applied on paper as a rigid one body and allowed to act as a water reservoir, releasing small amounts of water in a controlled way. Water acts as a cleaning agent, removing patinas, dust, as well as byproducts of cellulose hydrolysis and oxidation from paper. Gellan gel, in turn, is able to adsorb the soiling material, solubilized by water, performing a very effective and harmless (not invasive) cleaning task. When applied on CNC-treated samples, Gellan gel acts also as a CNC remover and carrier, delivering them through the flow system applied on it, to a suitable electrochemical detector (see the Experimental Section and Figure S1).

**Figure 3:**
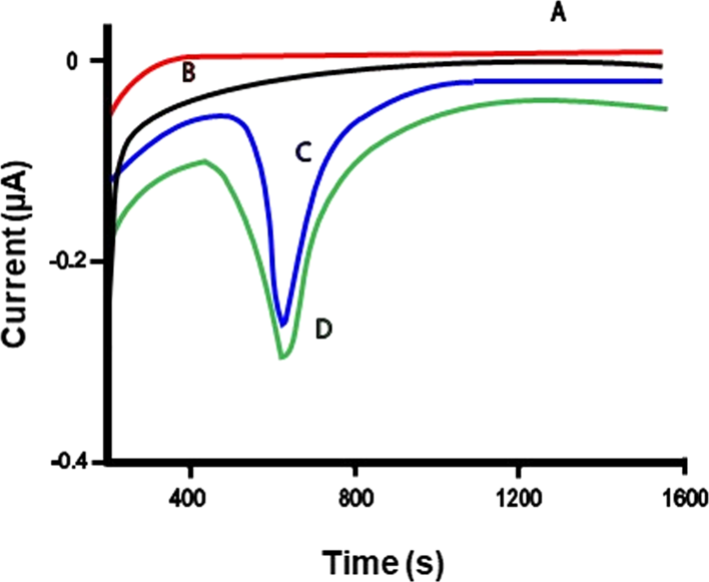
Current [μA] vs time [s] curves measured in amperometry experiments performed by means of the electrochemical monitoring tool described in the text.^38^ Traces: (A) buffer; and the amount of cellulose removed from (B) water pristine paper; (C) paper treated with N_CNCs; and (D) paper treated with S_CNCs. Applied potential: + 50 mV; measurements made in phosphate buffer at pH 6.8 + 0.1 M KCl.

As shown in Figure 3, these samples yielded the current peak appearance after 400 s from the application of Gellan hydrogel (interval time for the gel to absorb the nanocellulose, reach the bioreactor, and be digested into something that can be measured electrochemically, such as glucose), indicating the successful removal of the nanocrystals. In the absence of CNCs on the samples, this evident current peak is absent (in aged or non-aged Whatman paper, Figure 3) or has a very low intensity because it is caused by degradation products of cellulose in paper due to aging (see data on the Breviarium in the next paragraph). AFM topographies (Figure 2) as well as SEM images (Figure S6) supported this result as, after cleaning with gel, the coating on paper fibers due to CNCs completely disappeared, restoring the morphology of pristine paper. In particular, as shown by the AFM results acquired after cleaning and CNC removal process reported in Figure 2(d,e), the original texture of paper was apparently restored, with re-appearance of fibers featuring the same dimension as the starting sample. Interestingly, the surface roughness values, calculated from AFM topographies, were found to be 61 ± 7 nm and 52 ± 9 nm for the sample after removal of S_CNCs and N_CNCs, respectively, indicating that cleaning had quite probably removed additionally weakly bound material from the paper surface.

### Application of the Conservation Treatment to a Real Sample

**Figure 4:**
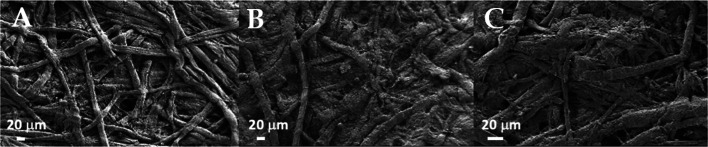
FE-SEM results of *Breviarium romanum ad usum fratrum minorum,* treated with (A) water; (B) S_CNCs; and (C) N_CNCs.

The last step of this study was the application of the CNC treatment on samples from a real aged book, *Breviarium romanum ad usum fratrum minorum* (1738), called, in the following, *Breviarium.* We had completely characterized that in our previous studies.^38−40^Figure 4 shows the FE-SEM results of a pristine *Breviarum* page (left image), a page treated with S_CNCs (middle), and a page treated with N_CNCs (right). The surface morphology appears more compact in samples treated with both, with S_CNCs coating showing a better film-forming ability, confirming what was observed on the Whatman samples and the coating properties of CNCs, while control water-treated samples presented pores and exposed fibers, which is less observed in the case of CNC-treated paper samples. Again, the smoother surface yielded by S_CNCs may indicate the higher penetration ability of N_CNCs among paper fibers, as hypothesized previously.

**Figure 5:**
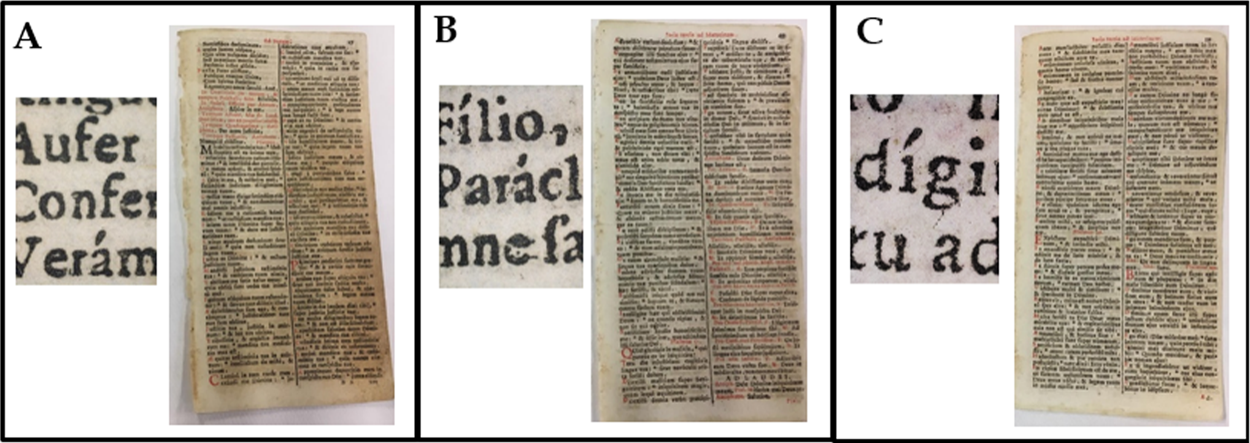
Images of pages and details from *Breviarium romanum ad usum fratrum minorum* treated with (A) water (pristine); (B) S_CNC; and (C) N_CNC.

At the same time, a visual analysis of the *Breviarium* pages treated with the two typologies of CNCs (reported in the Figure 5) demonstrates that the presence of transparent CNCs does not influence the features of the written parts, as the inked letters are clearly readable in all samples. Actually, as arguable from the results summarized in Table 3, the application of CNCs induces a slight improvement in the optical quality of paper (with respect to the untreated one) due the increase in the L* value and the concomitant decrease of a* and b* ones.

**Table 3:** Colorimetric Variations, pH, and Tensile Test Values of Breviarium Samples Treated with Water or with CNC Suspensions

paper	Δ*L*	Δ*a*	Δ*b*	Δ*E*	pH	maximum stress [MPa]	maximum strain [%]
treated with water					8.0 ± 0.1	7.7 ± 0.5	1.3 ± 0.1
untreated	–2.71	0.84	3.64	3.30 ± 0.09	7.8 ± 0.1	7.8 ± 0.8	1.4 ± 0.1
treated with S_CNC	1.71	–0.66	–2.75	3.30 ± 0.07	7.6 ± 0.2	7.3 ± 0.4	1.6 ± 0.2
treated with N_CNC	1.79	–0.43	–3.89	4.30 ± 0.08	8.1 ± 0.2	12 ± 1	2.2 ± 0.2

The application of the treatment to a real sample was very useful to definitively discriminate the behavior of sulfated from uncharged CNCs. Indeed, the presence of a sulfate group in the S_CNCs induced a slight worsening of the overall acidity and mechanical properties of paper. The acidity of S_CNCs, which did not have a significant impact on the pure cellulose Whatman paper, induced only a slight pH decrease from 8.0 ± 0.1 of pristine paper to 7.6 ± 0.2 of S_CNC-treated paper. In addition, the maximum stress did not change significantly (from 7.7 ± 0.5 to 7.3 ± 0.4 MPa). However, on longer aging times, the pH decreases induced by the surface sulfation of S_CNCs could increase the risk of cellulose hydrolysis, decreasing the polymerization degree of paper fibers and degrading the mechanical properties. Conversely, the strong reinforcement yielded by the N_CNCs treatment, with up to 50% enhancement of maximum stress and strain compared to the values of the pristine Breviarium paper, may be ascribed again to the penetration of the N_CNCs and effective coating of the individual fibers, resulting in improved bulk (or at least not limited to the paper surface layer) interfiber adhesion at the microscopic level and increased cohesivity at the macroscopic level. Furthermore, no pH variation was produced by the application of an N_CNCs protective treatment.

**Figure 6:**
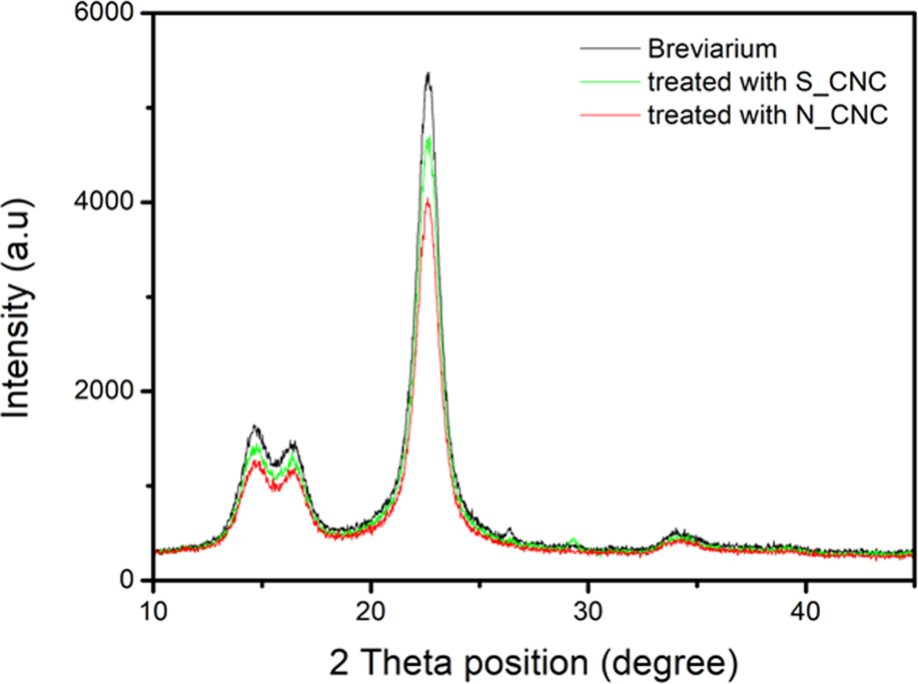
X-ray diffraction spectra acquired under glancing incidence conditions of pristine Breviarium page (gray line) and pages of Breviarium treated with N_CNCs (red line) and S_CNCs (green line).

XRD analysis, the results of which are reported in Figure 6, was performed under glancing incidence conditions, and it confirmed the negligible influence of the small nanocellulose mass fraction on the relevant spectra. The small variations in diffraction intensity yielded negligible variations (∼1%) in the C.I., detected by the Segal method, which was ∼88% in all three samples.

**Figure 7:**
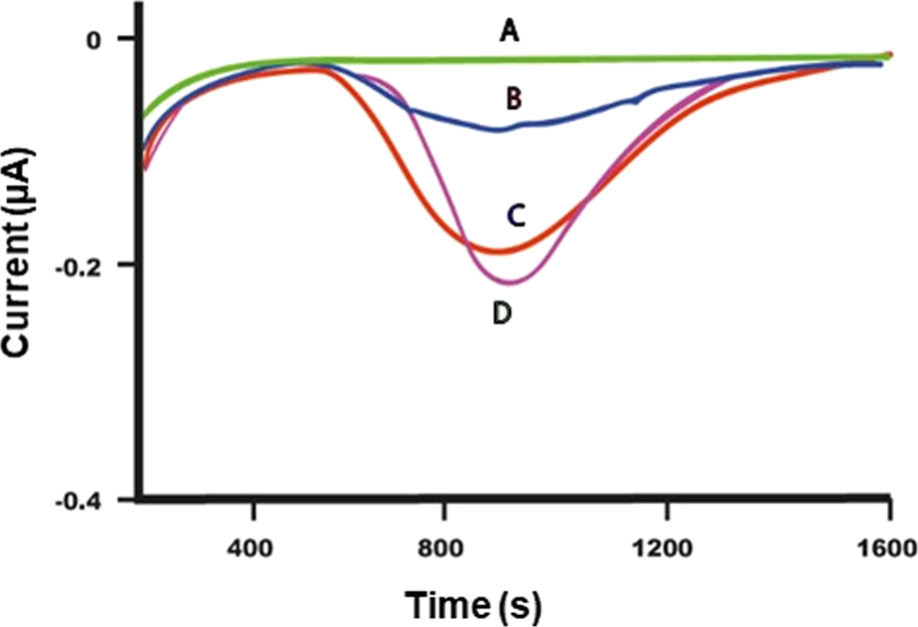
Current [μA] vs time [s] curves measured in amperometry experiments during the cleaning of paper samples. Trace: (A) buffer; and the cellulose amount removed from (B) pristine *Breviarium* page; (C) *Breviarium* page treated with N_CNCs; and (D) *Breviarium* page treated with S_CNCs. Applied potential: +50 mV; measurements made in PBS pH 6.8 + 0.1 M KCl.

As for the Whatman paper, also in the case of Breviarium, the removal of CNCs was followed by coupling of the electrochemical monitoring system with Gellan hydrogel. Figure 7 shows the amperometric plots monitoring the cleaning of the Breviarium pages, which was performed with Gellan gel applied on pristine pages (Figure 7 trace B) and those treated with N_CNCs (trace C) and S_CNCs (trace D). These samples yielded current peaks after 400 s from the application of Gellan hydrogel, indicating the effective removal of CNCs. The pristine page of the Breviarium yielded an analogous peak with very low intensity (trace B of Figure 7), caused by the removal of endogenous degraded cellulose present on the paper surface due to the natural aging of the page (due to the bad conservation state of Breviarium with respect to Whatman sample, where the same peak was absent). The amount of material removed from S_CNC- or N_CNC-coated samples was higher (traces D and C, respectively) with respect to that removed from the pristine page due to the nanocellulose removal by the gel. The FE-SEM investigation shown here is for the sample treated with N_CNC only, and it is shown in Figure S7; it supported the reversibility of nanocellulose coating.

## Conclusions

In this work, an innovative procedure to restore and reinforce, in a sustainable way, paper artworks is presented. The outstanding results reported here demonstrate that CNC application is compatible with paper materials, and, when applied on real aged samples, they can improve optical quality and mechanical properties. However, care should be taken in choosing the chemical surface derivatization method of CNCs, which could severely influence the effectiveness of the consolidation method. Indeed, the presence of surface sulfation suggests different aggregation properties in S_CNCs, when compared to N_CNC. Moreover, the functional group determines a difference in the consequence of aging. The pH of paper treated with S_CNCs decreases under accelerated aging, which means that it will negatively affect the conservation of a paper artifact treated with S_CNCs in the long term. This suggests that the use of S_CNCs for water protection would require a deacidifying additive to neutralize the excess acidity due to the sulfate functionalization. Conversely, N_CNCs are safer for paper conservation treatment because they do not compromise its pH and mechanical properties with aging. Moreover, for the first time, we demonstrated the reversibility of this restoration strategy by an easy and straightforward removal of the conservation treatment with Gellan hydrogel. The removal can be easily followed by interfacing of an efficient real-time monitoring electrochemical tool to the hydrogel, which allows us to remove and detect the removal of the nanocrystals at approximately 400 s after the application of the gel. This work represents a fundamental study on the interaction of CNCs with aged and non-aged paper materials.

## Authors Contribution

The manuscript was written through contributions of all authors. All authors have given approval to the final version of the manuscript. L.M. and C.M. equally contributed to this work as corresponding authors.

## Supporting Information Available

Electrochemical monitoring tool, XRD spectra and AFM images of CNCs, FTIR, data of untreated and CNC-treated paper samples, SEM images of CNC-treated samples uncleaned and cleaned with Gellan gel, and elemental analysis of samples under study (PDF)

## Abbreviations

CNC: cellulose nanocrystals
S_CNC: sulfate CNC
N-CNC: neutral CNC
FTIR: Fourier Transform InfraRed
C.I.: crystallinity index
XRD: X-ray diffraction
SEM: scanning electron microscopy
HPLC: high performance liquid chromatography.
