# Supplementary material for: Toward a Reversible Consolidation of Paper Materials Using Cellulose Nanocrystals
Source: ACS Appl Mater Interfaces. 2021 Sep 14;13(37):44972–82. doi: 10.1021/acsami.1c15330 (PMC8461603; doi:10.1021/acsami.1c15330)
Supplement: Supplementary file 1 — am1c15330_si_001.pdf [file am1c15330_si_001.pdf]

Supplementary Information for Publication

Towards a reversible consolidation of paper materials using cellulose nanocrystals

Alessandra Operamolla,[†] Claudia Mazzuca,*[§¶] Laura Capodieci,[‡] Francesca Di Benedetto,[‡] Leonardo Severini[¶], Mattia Titubante,[¶] Andrea Martinelli,[£] Valter Castelvetro,[†] and Laura Micheli*[§¶]

[†] Dipartimento di chimica e Chimica Industriale, Università di Pisa, via Giuseppe Moruzzi 13, I-56124, Pisa, Italy

[§] Dipartimento di Scienze e Tecnologie Chimiche, Università degli Studi di Roma Tor Vergata, Via della Ricerca Scientifica, I-00173 Rome, Italy

[¶] Unità CSGI (Consorzio Interuniversitario per lo Sviluppo dei Sistemi a grande Interfase) di Roma Via della Ricerca Scientifica, I-00173 Rome, Italy

[‡] Laboratory for Functional Materials and Technologies for Sustainable Applications (SSPT-PROMAS-MATAS), ENEA – Italian National Agency for New Technologies, Energy and Sustainable Economic Development, S.S. 7 Appia km 706, I-72100 Brindisi (Br), Italy

£ Dipartimento di Chimica, Università degli Studi di Roma " Sapienza" - Piazzale Aldo Moro 5, 00185 Roma

* LM and CM equally contributed to this work as corresponding authors.

LM: laura.micheli@uniroma2.it. CM:claudia.mazzuca@uniroma2.it

EXPERIMENTAL SECTION

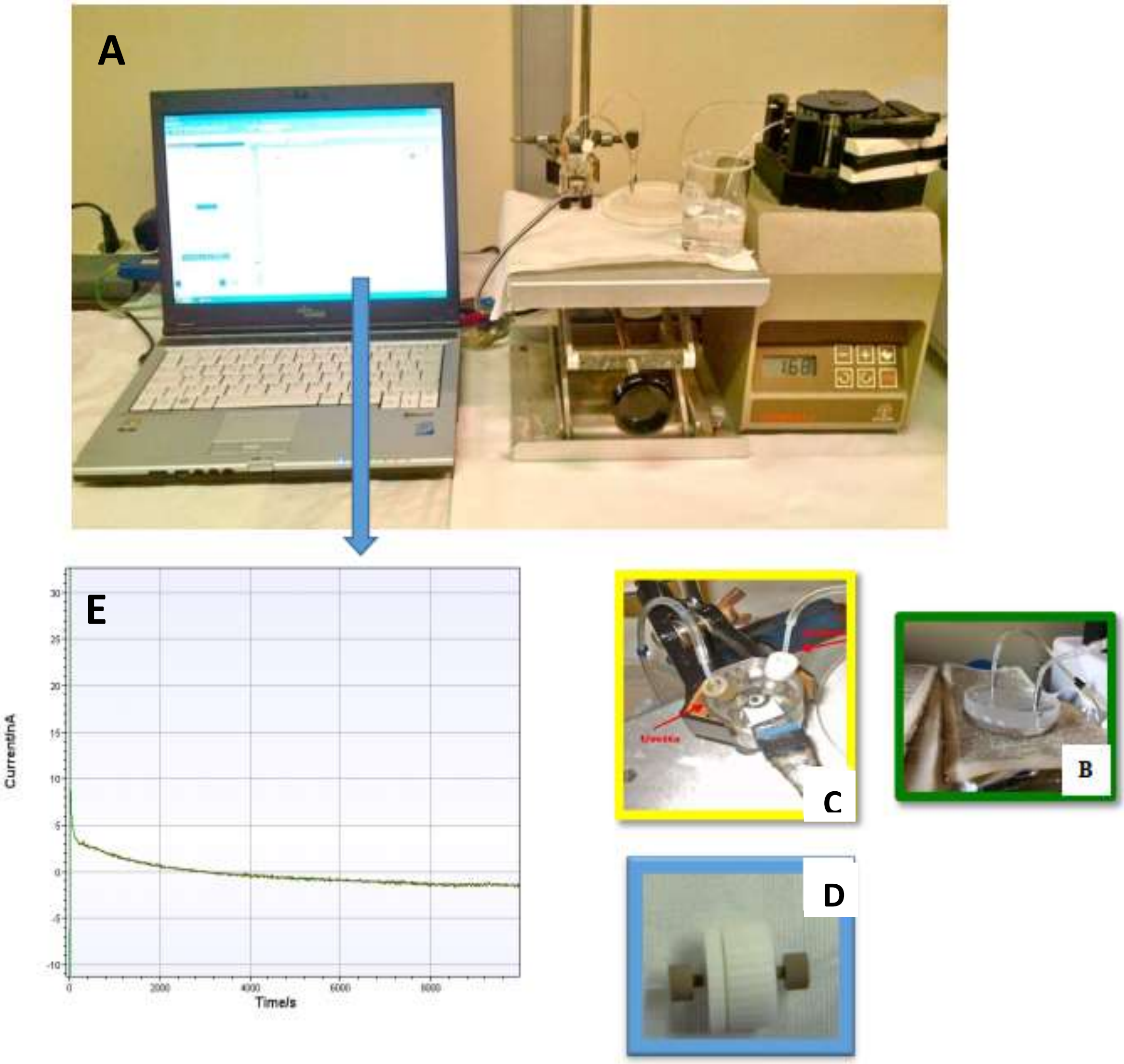

Figure S1. *(upper panel):* monitoring tool for the detection of endogenous glucose due to the degradation of cellulose chain of paper and the removal of nanocellulose film form the paper artworks using Gellan gel; A) monitoring tool plus connected to laptop; B) sampling plate on *Breviarium* page, C) screen printed electrode in thin layer cell; D) bioreactor; E) amperometry graph: current *vs* time.

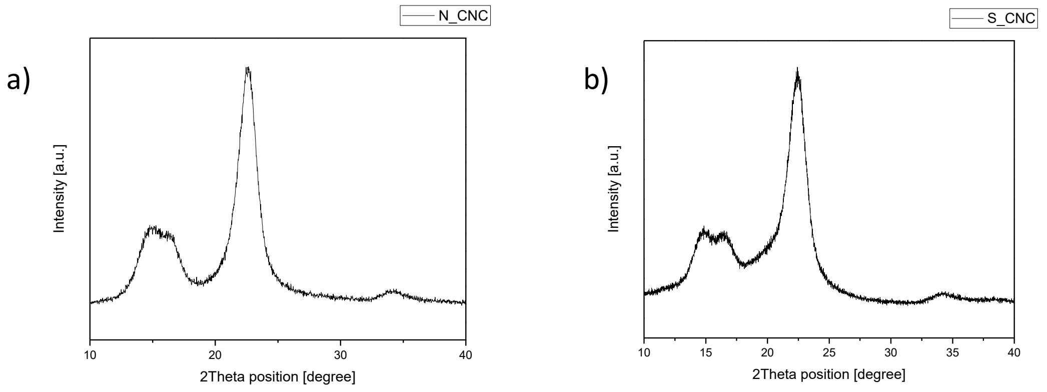

Figure S2. XRD spectra of pure a) N_CNCs and b) S_CNCs.

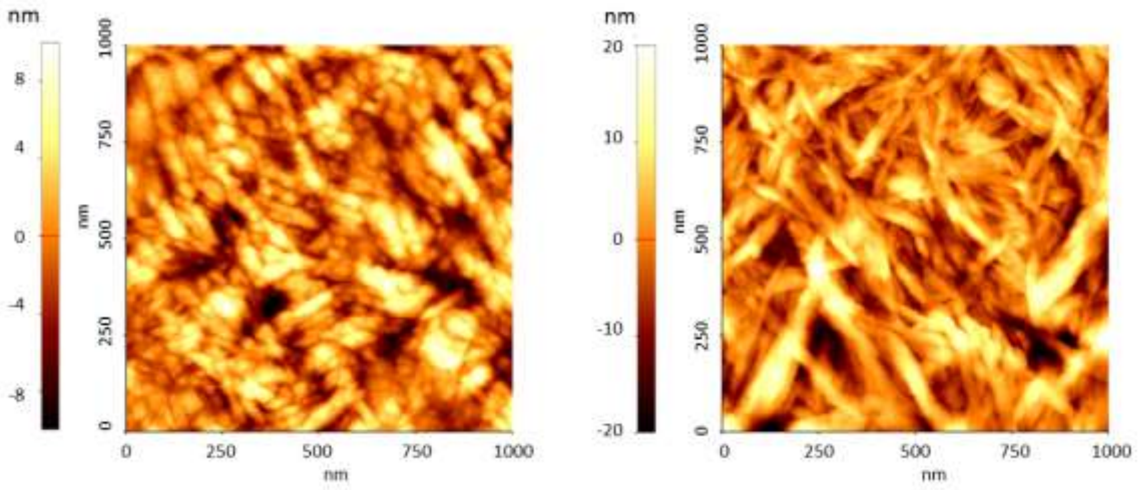

Figure S3 1 x 1 µm^2 AFM non-contact topographies of S_CNC *(left)* and N_CNC *(right)*. The AFM topographies certify the presence of rod-like nanocrystals.

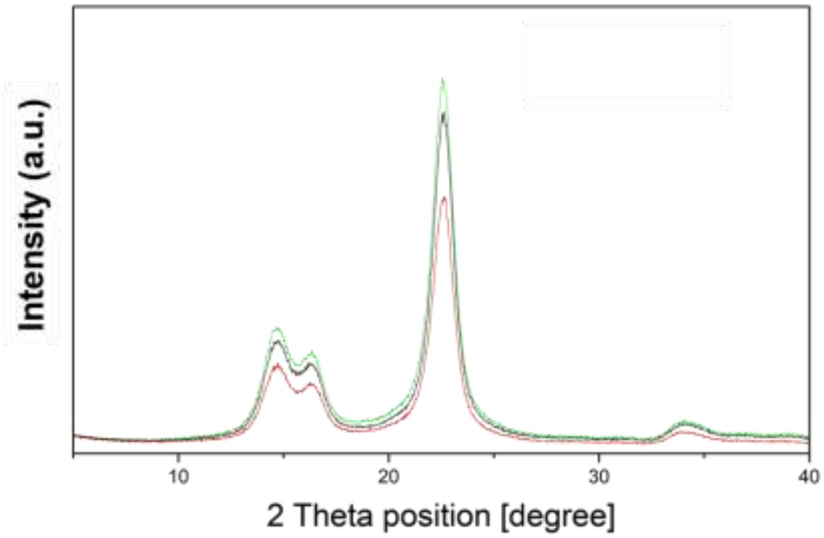

Figure S4. XRD spectra on untreated *(green line)* and treated with N_CNC *(gray line)* or S_CNC *(red line)* unaged paper samples

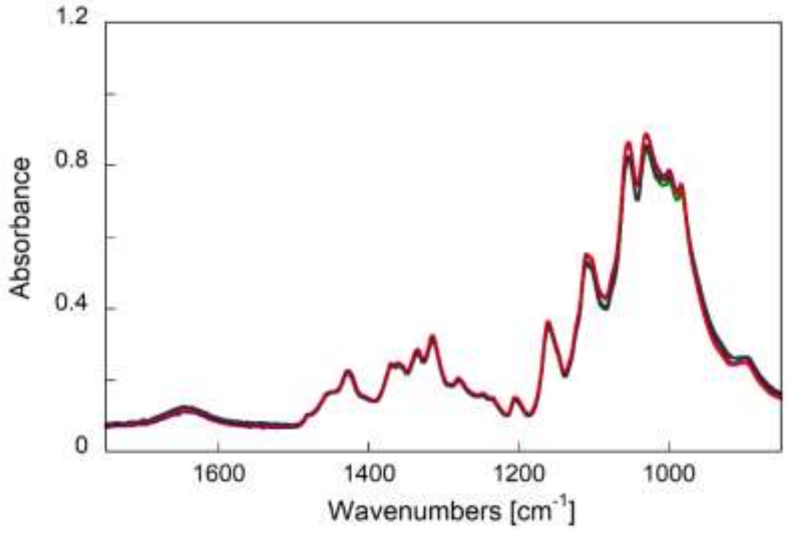

Figure S5. FTIR spectra of: pristine *(red line);* treated with N_CNC *(gray line);* or S_CNC *(green line)* unaged paper samples.

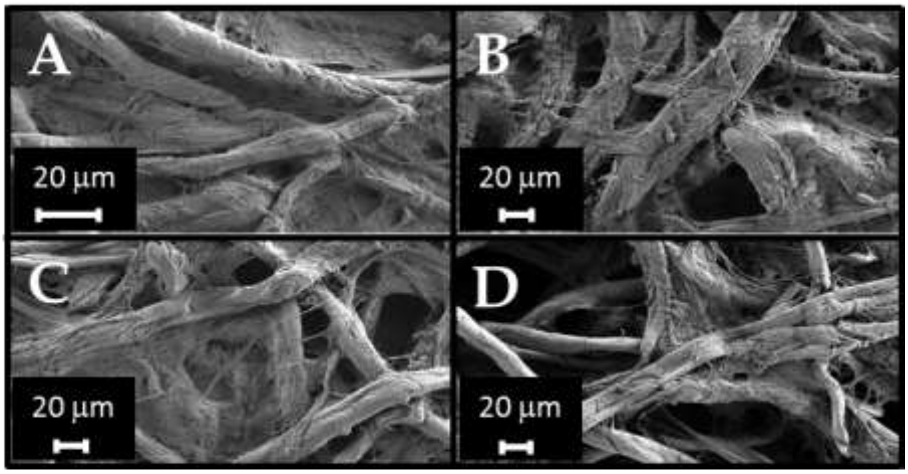

Figure S6. SEM images of paper samples treated with (A): N_CNC; (B): S_CNC; and of samples A and B after cleaning with Gellan gel (C and D, respectively).

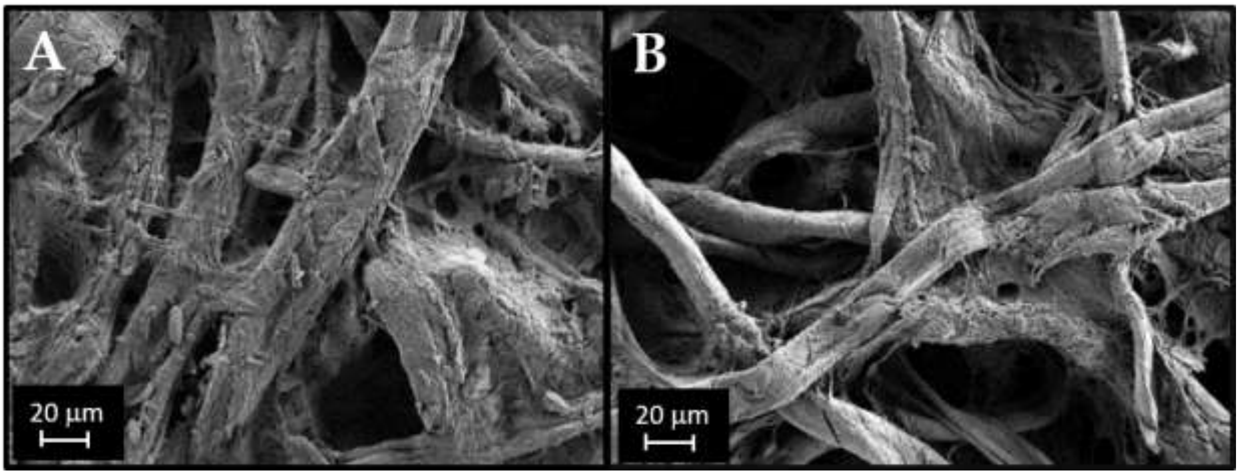

Figure S7. FE-SEM micrographs of (A): a *Breviarium* page treated with N_CNC; (B): a *Breviarium* page treated with N_CNC after the cleaning with Gellan gel. Scale bar 20 µm for both micrographies.

Table S1. Cristallinity Index (C.I.) calculated from FT-IR and XRD.

Sample	C.I. calculated from FT-IR	C.I.[a] calculated from XRD
S_CNCs		
N_CNCs		
Whatman™ pristine	0.64± 0.03	0.89
Whatman™ with S_CNC	0.65± 0.03	-
Whatman™ with N_CNC	0.65± 0.03	-
Whatman™ aged	0.59± 0.03	0.90
Whatman™ with S_CNC, and aged	0.64± 0.03	0.91
Whatman™ with N_CNC, and aged	0.64± 0.03	0.91
Breviarium, pristine		0.88
Breviarium treated with S_CNC		0.88
Breviarium treated with N_CNC		0.86

[a] XRD was carried out on S_CNC and N_CNC samples prepared as thin films in glancing incidence conditions. This kind of set-up and sample preparation was not the ideal one for measuring the C.I. of the CNCs, as it was chosen to detect any relevant variation on the surface of paper (that was the objective of our investigation).

Elemental analyses

Elemental analyses were performed by the use of a Carlo Erba EA 1108 CHNS Elemental analyser or of a Elementar Vario Micro Cube analyzer. Data reported in **Table S2** are relevant to S_CNC and N_CNC, analysed as 5 mg samples on the Micro Cube instrument, in order to detect the amount of sulfur and calculate the degree of substitution in S_CNC.

Data reported in the **Table S3** are relevant to all samples analysed in this work and were carried out with the EA 1108 instrument.

Table S2. Elemental combustion analyses of cellulose nanocrystals. The data in the following Table were acquired on an Elementar Vario Micro Cube analyzer on 5 mg samples.

Sample	C [%][a]	H [%]	S [%]	O [%][a]
S_CNCs	40.35	5.89	0.88	52.88
N_CNCs	40.83	6.52	0	52.65

For calculating the degree of sulfation of S_CNC, we derived the average molecular formula (1) for S_CNC by dividing each percentage by the atomic mass of each element and the Avogadro's constant:

$$C_{3.36}H_{5.84}O_{3.31}S_{0.0274} \qquad (1)$$

At this point we considered that for each mole of S in the sample an -$OSO_3^-\cdot H_3O^+$ or -OSO_3H group should be chemically linked to the C(6) of one glucopyranose unit. Therefore 0.0274 x 4 moles of oxygen should belong to sulfated substituents. In this way the molecular formula became:

$$C_{3.36}H_{5.84}O_{3.22}(SO_3)_{0.0274} \qquad (2)$$

Now the formula was multiplied by 1.79 in order to normalize the moles of carbon in the molecular formula to 6:

$$C_6H_{10.45}O_{5.84}(SO_3)_{0.049} \qquad (3)$$

Now the excess oxygen with respect to the molecular formula of cellulose ($C_6H_{10}O_5$) was attributed to crystallized water in the sample. Therefore, the new molecular formula became (4):

$$C_6H_9O_5(SO_3)_{0.049}\cdot(H_2O)_{0.84} \qquad (4)$$

Supposing an overestimation of oxygen due to the presence of traces of other elements or the presence of an error on H percentage detection (justifying the value of 9 found for hydrogen instead of 10), based on the results of elemental analyses, S_CNC were composed of pure cellulose with a surface sulfation with a substitution degree (DS) of ~0.05. The value of pH found for the relevant S_CNC suspensions (immediately after dialysis and before freeze-drying) of 3.94, would suggest that the sulfated groups were present as $-OSO_3^{-}\cdot H_3O^{+}$ in the freeze-dried sample.

Table S3. Elemental combustion analyses of cellulose nanocrystals, pristine Whatman paper and same paper after ageing, after ageing and treatment with CNCs, after ageing, treatment with CNCs and removal of the treatment via gellan gel and Cellulase treatment. The data in the following table were acquired on a Carlo Erba EA 1108 CHNS Elemental analyser.

Sample	N [%]	C [%]	H [%]	O [%][a]
S_CNCs	0.32	40.17	8.74	50.77
N_CNCs	0.19	41.45	8.28	50.08
Pristine Whatman paper	0.18	41.88	8.33	49.61
Aged Whatman paper	0.25	42.48	8.64	48.63
Aged Whatman paper after S_CNC treatment	0.20	42.70	8.56	48.54
Aged Whatman paper after N_CNC treatment	0.15	41.97	8.61	49.27
Aged Whatman paper after S_CNC treatment and after cleaning	0.13	42.13	8.57	49.24
Aged Whatman paper after N_CNC treatment and after cleaning	0.11	41.81	8.60	49.48

[a] Calculated elemental analyses outcomes are influenced by the water content.
